# Epidemiology and genetic characterization of influenza viruses circulating in Bhutan in 2022

**DOI:** 10.1371/journal.pone.0304849

**Published:** 2024-09-17

**Authors:** Kunzang Dorji, Chonticha Klungthong, Tshering Dorji, Tandin Wangchuk, Pema Yuden, Tshering Pelki, Tara Devi Ghishing, Govinda Gyemiry, Sonam Gyeltshen, Piyawan Chinnawirotpisan, Wudtichai Manasatienkij, Sonam Wangchuk, Aaron Farmer

**Affiliations:** 1 National Influenza Centre, Royal Centre for Disease Control, Ministry of Health, Thimphu, Bhutan; 2 Department of Virology, Armed Forces Research Institute of Medical Sciences, Bangkok, Thailand; 3 ICT, Royal Centre for Disease Control, Ministry of Health, Thimphu, Bhutan; 4 Royal Centre for Disease Control, Ministry of Health, Thimphu, Bhutan; Lagos State University, NIGERIA

## Abstract

**Introduction:**

Influenza (Flu) causes considerable morbidity and mortality globally, and in Bhutan, Flu viruses are a leading cause of acute respiratory infection and cause outbreaks during Flu seasons. In this study, we aim to analyze the epidemiology and the genetic characterization of Flu viruses circulated in Bhutan in 2022.

**Method:**

Respiratory specimens were collected from patients who meet the case definition for influenza-like illness (ILI) and severe acute respiratory infection (SARI) from sentinel sites. Specimens were tested for Flu and SARS-CoV-2 viruses by RT-PCR using the Multiplex Assay. Selected positive specimens were utilized for Flu viral genome sequencing by next-generation sequencing. Descriptive analysis was performed on patient demographics to see the proportion of Flu-associated ILI and SARI. All data were analyzed using Epi Info7 and QGIS 3.16 software.

**Result:**

A weekly average of 16.2 ILI cases per 1000 outpatient visits and 18 SARI cases per 1000 admitted cases were reported in 2022. The median age among ILI was 12 years (IQR: 5–28) and SARI was 6.2 (IQR: 2.5–15) years. Flu A(H3N2) (70.2%) subtype was the most predominant circulating strain. Flu A(H1N1)pdm09 and Flu B viruses belonged to subclades that were mismatched to the vaccine strains recommended for the 2021–2022 season but matched the vaccine strain for the 2022–2023 season with vaccine efficacy 85.14% and 88.07% respectively. Flu A(H3N2) virus belonged to two subclades which differed from the vaccine strains recommended in both the 2021–2022 and 2022–2023 seasons with vaccine efficacy 68.28%.

**Conclusion:**

Flu virus positivity rates were substantially elevated during the Flu season in 2022 compared to 2021. Flu A(H3N2) subtype was the most predominant circulating strain in the country and globally. Genetic characterization of the Flu viruses in Bhutan showed a close relatedness of high vaccine efficacy with the vaccine strain that WHO recommended for the 2022–23 season.

## Introduction

Influenza (Flu) is a major cause of respiratory infections in humans, resulting in significant morbidity and mortality worldwide and infecting 5–15% of the global population yearly [1]. Flu infections contribute to considerable morbidity and mortality globally in both temperate and tropical regions, causing 3–5 million cases of severe illness and 250,000–650,000 deaths annually [1, 2]. All age groups can be affected by Flu, however, pregnant women, children under 59 months, the elderly aged above 65 years, individuals with chronic medical conditions, individuals with immunosuppressive conditions, and healthcare workers are at higher risk of severe illness [3, 4].

Genetic characterization of Flu viruses is essential for understanding their epidemiology, evolution, pathogenicity, antigenicity, and efficacy of vaccines and therapeutics. The ability to monitor the evolution of Flu viruses, provide optimal data to inform the biannual World Health Organization (WHO) recommendations for seasonal Flu vaccines, assess antiviral susceptibility, and update diagnostic reagents and protocols as and when necessary is vital in strengthening the network of surveillance and response capacity of WHO member states. Genetic information also provides crucial granularity for epidemiological studies and the development of future treatments enabling improved surveillance, diagnosis, prevention, and control of Flu [5].

In Bhutan, acute respiratory infection (ARI) continues to be one of the top 10 diseases reported annually for the last ten years in the National Early Warning Alert and Response Surveillance System (NEWARS) [6]. A previous study reported an incidence of Flu-associated hospitalizations in all age groups of 50/100,000 persons in 2015 and 118/100 000 persons in 2016 with the highest rates among children aged below 5 years: 182/100 000 in 2015 and 532/100 000 in 2016 [7].

Flu-associated infection has contributed to a substantial burden of severe illness requiring hospitalization, especially among children and older adults [7]. Due to the significant burden, the Ministry of Health of Bhutan introduced the seasonal Flu vaccine into routine immunization service in 2019 for all high-risk groups recommended by the Strategic Advisory Group of Experts on Immunization (SAGE). To help understand the dynamic of Flu viruses in severe respiratory disease and the potential impact of improved vaccination and other countermeasures, it is important to study the genetic characterization of circulating Flu viruses. To this goal, this paper describes the epidemiological and genetic characterization of Flu A(H1N1)pdm09, Flu A(H3N2), and Flu B viruses circulated in Bhutan in the year 2022.

## Method

### Study design and setting

**Fig 1:**
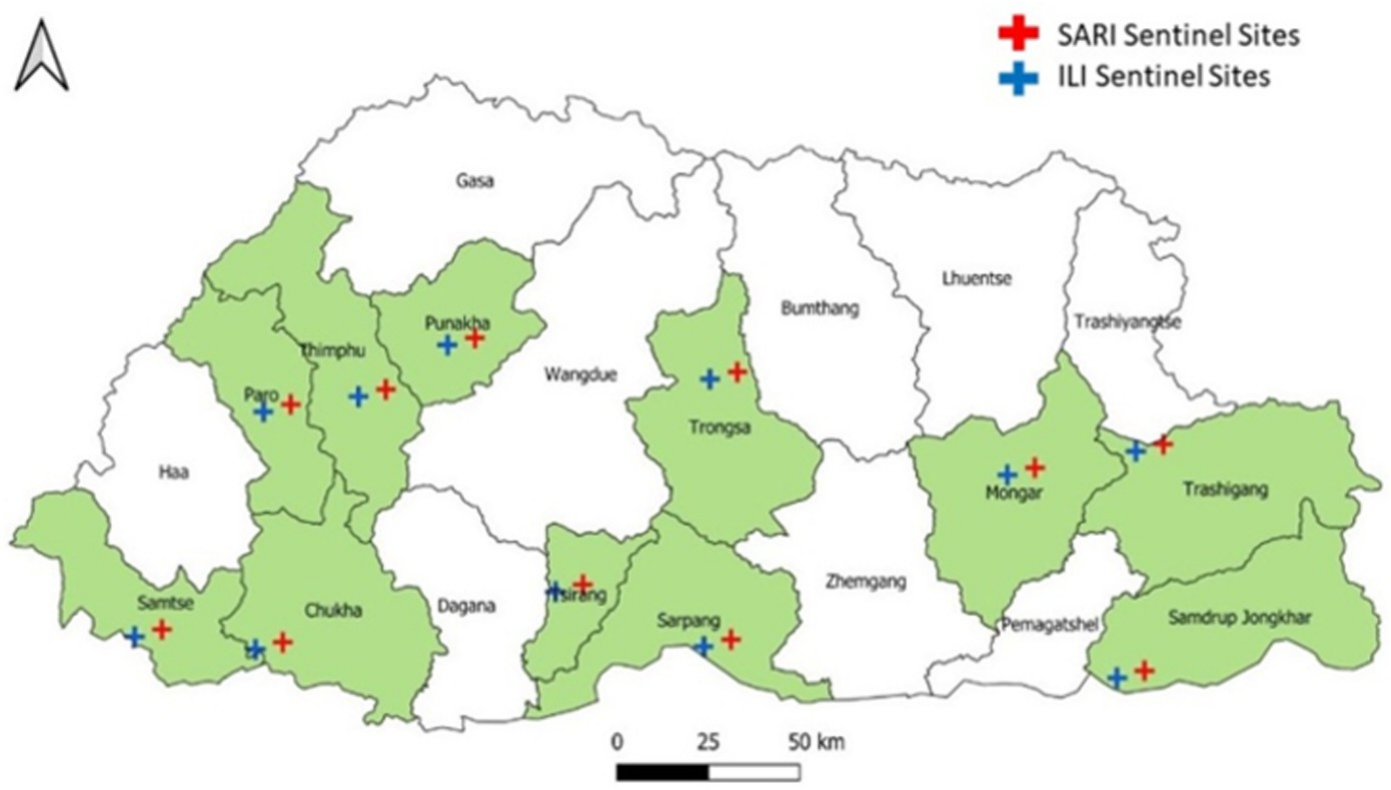
Influenza-like illness (ILI) and severe acute respiratory infection (SARI) sentinel surveillance hospitals.

This study is based on prospective sentinel surveillance for Flu and SARS-CoV-2 as per the COVID-19 integrated influenza surveillance guideline developed in 2022 [8]. There are eleven Flu sentinel hospitals: Jigme Dorji Wanghck National Referral Hospital, Thimphu, Punakha Hospital, Paro Hospital, Monggar Regional Referral Hospital, Phuntsholing Hospital, Gelephu Regional Referral Hospital, Trongse Hospital, Tsirang Hospital, Trashigang Hospital, Samtse Hospital and Samdrup Jongkhar Hospital. The sentinel sites were selected based on population, patient referral, geographical locations, and climatic conditions representation (Fig 1).

### Participants and sample size

As per the guideline, influenza-like illness (ILI) cases were enrolled randomly for sample collection (atleast 5–10 samples per week or 40 samples per month) representing all age groups. For severe acute respiratory infection (SARI) cases, all patients meeting the criteria were recommended for specimen collection [8]. The recruitment for this study was started from January 1 to December 31, 2022.

Nasal/throat swab specimens were collected from patients meeting the case definition for ILI (ILI case definition: Any person with acute respiratory infection with; Fever ≥ 38°C, Cough, AND Onset within the last 10 days), and SARI (SARI Case definition: Any person with acute respiratory infection with; History of fever or fever ≥ 38°C, AND Cough, AND With onset within the last 10 days, AND Require hospitalization) along with clinical and epidemiological information through a structured interviewer questionnaire. Written Informed consent was obtained for sample collection for all three categories of patients: adult consent for 18 years and above, assent consent for 7–17 years, and parental/guardian consent for 6 years and below.

### Data collection

All demographics including clinical and laboratory information were collected through a structured surveillance questionnaire, verified, and entered into the COVID-19 Integrated Influenza surveillance system.

### Data analysis

Surveillance data were extracted from the COVID-19 Influenza Integrated surveillance system. All data were analyzed using Epi Info7 and QGIS 3.16 was used to generate the mapping distribution of the Influenza positivity in the country. Descriptive statistics, such as proportions and medians with interquartile ranges, were used to present the distribution of age, sex, and clinical symptoms among influenza and SARS-CoV-2 positive ILI and SARI cases.

### Laboratory detection

#### Influenza SARS-CoV-2 (Flu SC2) multiplex assay

Specimens were tested for Flu viruses and SARS-CoV-2 by RT-PCR using the US-CDC Influenza SARS-CoV-2 (Flu SC2) Multiplex Assay. Viral RNA was extracted from 200 μl of Throat/Nasal swabs collected in viral transport media using Spin-X Viral RNA Extraction Kit (SD Biosensor, South Korea) and KingFisherTM Flex Purification system (Thermo Fisher, India) following the manufacturer’s instructions. The extracted viral RNA was subjected to US-CDC Flu SC2 Multiplex Assay. A total of 5 μl of viral RNA was added to the tube containing the mixture of 7.75 μl of nuclease-free water, 3 μl of Flu SC2 combined primer mix, 3 μl of Flu SC2 combined probe mix and 6.25 μl TaqPathTM 1-Step Multiplex Master Mix (No ROX).

Subsequently, the cycling condition was set at 1 cycle of reverse-transcription step at 20°C for 2 minutes and 50°C for 15 minutes followed by 1 cycle of pre-denaturation at 95°C for 3 minutes followed by 45 cycles of 95°C at 15 seconds, 55°C for 33 seconds. Cycle threshold (CT) value <40 for Flu A, Flu B, SC2 and <35.00 CT for Ribonuclease P (RNase P or RP) was considered positive whereas, CT value ≥40.00 for Flu A, Flu B, SC2 and ≥35.00 CT for RP were considered negative. For each protocol, positive and negative controls were run in each test to validate the test result. Furthermore, samples that tested positive for Flu A and/or B virus were subtyped and lineage determined using the Human Influenza Virus Real-Time RT-PCR Diagnostic Influenza A/B Typing Kit (US-CDC) based on the manufacturer’s instruction.

#### Influenza viral genome sequencing

Selected representative Flu positive specimens were utilized for Flu viral genome sequencing by following the “Sequencing First” strategy using next-generation sequencing (NGS) [9]. NGS experiments were performed at the World Health Organization Collaborating Centre (WHO CC), US-CDC, and the Department of Virology, Armed Forces Research Institute of Medical Sciences (AFRIMS-Virology). Flu genomic amplicon sequencing protocols previously described by Zhou et al., 2009 and 2014 were performed for Flu A and Flu B viral genome sequencing, respectively [10, 11]. The SuperScript™ III One-Step RT-PCR System with Platinum™Taq High Fidelity DNA Polymerase (Thermo Fisher Scientific) was used to generate multiplex RT-PCR amplicons. PCR products containing Flu viral genomic segments were visualized using the QIAxcel Advanced System (QIAGEN). Obtained PCR products underwent purification using Agencourt AMPure XP Beads, following the manufacturer’s instructions. Subsequently, purified PCR products were sequenced using the Illumina MiSeq platform. Paired-end libraries were prepared using the Illumina DNA Library Prep Kit (Illumina) and the Nextera CD Index Kit (Illumina). Sequencing reactions were performed using a 500-cycle (2 × 250-bp, paired-end) MiSeq v2 Reagent Kit (Illumina), following the manufacturer’s instructions.

#### Viral genome sequence data analysis

The obtained paired-end reads were subjected to quality trimming using BBtools v.37.62 (Bushnell, 2018) with the following criteria: base quality threshold of > 25, minimum length requirement of 100, and minimum average quality threshold of 20 [12]. The genome consensus sequence of each sample was derived by aligning the trimmed reads to an appropriate reference genome sequence selected from the GenBank database (S1 File: Gene Accession Number). This alignment was performed using BWA-MEM v.0.7.17 (Li, 2013) and iVAR v1.3.1 (Grubaugh et al, 2019), with the following criteria: mapping quality threshold > 30, base quality > 30, and a minimum depth of coverage of 10 [13, 14]. Ambiguous bases were identified and confirmed by genome-guided assembly using Trinity v2.14.0 (Grabherr, et al., 2011) [15]. All obtained Flu viral consensus genome sequences were submitted to the publicly accessible EpiFlu^TM^ database that is part of the Global Initiative on Sharing All Influenza Data (GISAID: http://www.gisaid.org) [16, 17].

For phylogenetic analysis, HA sequences of reference clades and vaccine strains were downloaded from EpiFlu^TM^ database (Accession Numbers provided in S2 File) and aligned using the Multiple Alignment using Fast Fourier Transform (MAFFT v7.310) [18], with separate alignments for each subtype. Maximum-likelihood trees were computed from each alignment using IQ-TREE v2.0.3 [19], with substitution models HKY+F+I for Flu A(H1N1)pdm09 and Flu B and TVM+F+I for Flu A(H3N2), and 1,000 ultrafast bootstrap replicates. The tree visualization was conducted utilizing either figtree v1.4.4 (http://tree.bio.ed.ac.uk/software/figtree/) or MEGA11 [20, 21].

#### Prediction of vaccine efficacy

Antigenic relatedness estimates were used to predict vaccine efficacy using the *P*_epitope_ model [22] which considers the antigenic sites variations between circulating viral strains and the vaccine strain by analysing epitope sites. This study compared the dominant epitope sequences of the HA1 domain, which plays a critical role in viral entry into host cell [23], in influenza A and B strains present in Bhutan from January to December 2022 with those of the 2022 influenza vaccines strain. Antigenic relatedness was then utilized to predict the vaccine efficacy (VE) base on the following formulas: E = 0.53–1.19 x *P*_epitope_ for A(H1N1)pdm09 [24], E = 0.47–2.47 x *P*_epitope_ for Flu A(H3N2) [25] and E = 0.6824–0.864 x *P*_epitope_ for Flu B [26].

#### Antigenic characterization

Antigenic characterization was conducted using hemagglutination-inhibition (HI) assays or a neutralization-based assay (High-content Imaging-based Neutralization Test (HINT)) for A(H3N2) with panels of post-infection ferret antisera to a subset of cell-propagated viruses [27]. Selection for antigenic characterization is based on the genetic characteristics of the virus (i.e., representation of every co-circulating genetic group), lack of mutations during cell-culture propagation, and date of collection (the recent collection is favored).

#### Antiviral drug resistance assays

A phenotypic assay was performed to assess the susceptibility of 11 Flu A(H3N2) virus isolates to neuraminidase (NA) inhibitors (oseltamivir, laninamivir, peramivir, and zanamivir) and the PA cap-dependent endonuclease inhibitor baloxavir. Representative samples for the antiviral assay were selected based on the criteria used for the antigenic characterization. We also investigated mutations associated with antiviral drug resistance in the NA gene sequences of Flu viruses obtained from this study. These mutations included the following; for Flu A(H1N1)pdm09: H275, for Flu A(H3N2): E119, D151, R292, N294, or Flu B: E117, R150, D197, I221, S249, R374, H273, G407 [28–30].

#### Ethical statement

This study was part of an influenza control program evaluation and the study protocol is being reviewed and approved annually by the Research Ethics Board of Health (REBH) in Bhutan:

Ref. No. REBH/Approval/2009/012 Approval Date: 30/03/2022. Patient written consent was obtained before sample collection, and parental or guardian consent was obtained for children below 7 years. Patient privacy was protected, and only anonymized data were used for the analysis.

## Results

### Epidemiological characteristics

A total of 4,287 ILI cases and 651 SARI cases were reported for the year with a weekly average of 16.2 ILI cases per 1000 OPD visits and 18 SARI cases per 1000 admitted cases from the sentinel hospitals. A total of 2,015 nasal/throat swab specimens were collected of which 1273 (63.2%) were ILI, 473 (23.5%) were SARI, 169 (8.4%) were ARI outbreak samples, and 100 (5.0%) specimens were received from non-sentinel hospitals (Table 1).

**Table 1:** Number of Flu specimens received and tested in 2022.

Sentinel Sites/ Hospitals/ Non sentinel Sites	ILI	SARI	Non sentinel sites	ARI Outbreak Samples	Grand Total
Samtse Hospital	316	48			364
Trongsa Hospital	268	21			289
Phuentsholing	118	44		5	167
Tsirang Hospital	113	9			122
Punakha Hospital	100	5		28	133
Samdrupjongkhar	98	18			116
Paro Hospital	93	104		23	220
Monggar ERRH	63	12			75
JDWNRH	43	92			135
Gelephu CRRH	31	120			151
Trashigang Hospital	30	0		6	36
Chhukha BHU I			3		3
Chhuzergang BHU			1		1
Gedu Hospital			13		13
Gomtu Hospital			1		1
Gyalposhing BHU I			1		1
Haa Hospital			1		1
Lungtenphu			76		76
RCDC			2		2
Wangdue Hospital			2		2
Lhamiozingkha Hospital				92	92
Trashi Yangtse Hospital				15	15
Grand Total	1273	473	100	169	2015

The median time taken to receive a Flu specimen to the nearest PCR testing lab was 6 days (IQR: 2–10 days), while the median time taken for testing was 2 days (IQR: 1–3) (Table 2). All specimens were tested for Flu viruses and SARS-CoV-2 by using Flu SC2 Multiplex assay and overall Flu positive was 15.2% (280/1846), SARS-CoV-2 positive was 4.0% (74/1846) and coinfection was 0.3% (5/1846). The proportion of Flu A(H3N2) (70.2%) subtype was the most predominant circulating strain, followed by SARS-CoV-2 (20.6%) and Flu A(H1N1)pdm09 (4.7%) subtype (Table 2).

**Table 2:** Sample processing turn-around time and proportion of Flu subtypes.

Variables	n (%)
Time Taken for Receive Samples (Days)	
Median (IQR)	6 (2–10)
Time Taken for Testing	
Median (IQR)	2 (1–3)
Dewathang Hospital	4 (2–7)
Gelephu CRRH	2 (0–5)
Monggar ERRH	6 (3–18)
Phuentsholing Hospital	3 (1–4)
Royal Centre for Disease Control	2 (1–3)
Influenza Subtypes and COVID-19	
Flu A/H3N2	252 (70.2)
Flu A/H3 and SARS-CoV-2	5 (1.4)
Flu A/Not Subtyped	2 (0.6)
Flu A(H1N1)Pdm09	17 (4.7)
Flu B/Victoria	9 (2.5)
SARS-CoV-2	74 (20.6)

**Fig 2:**
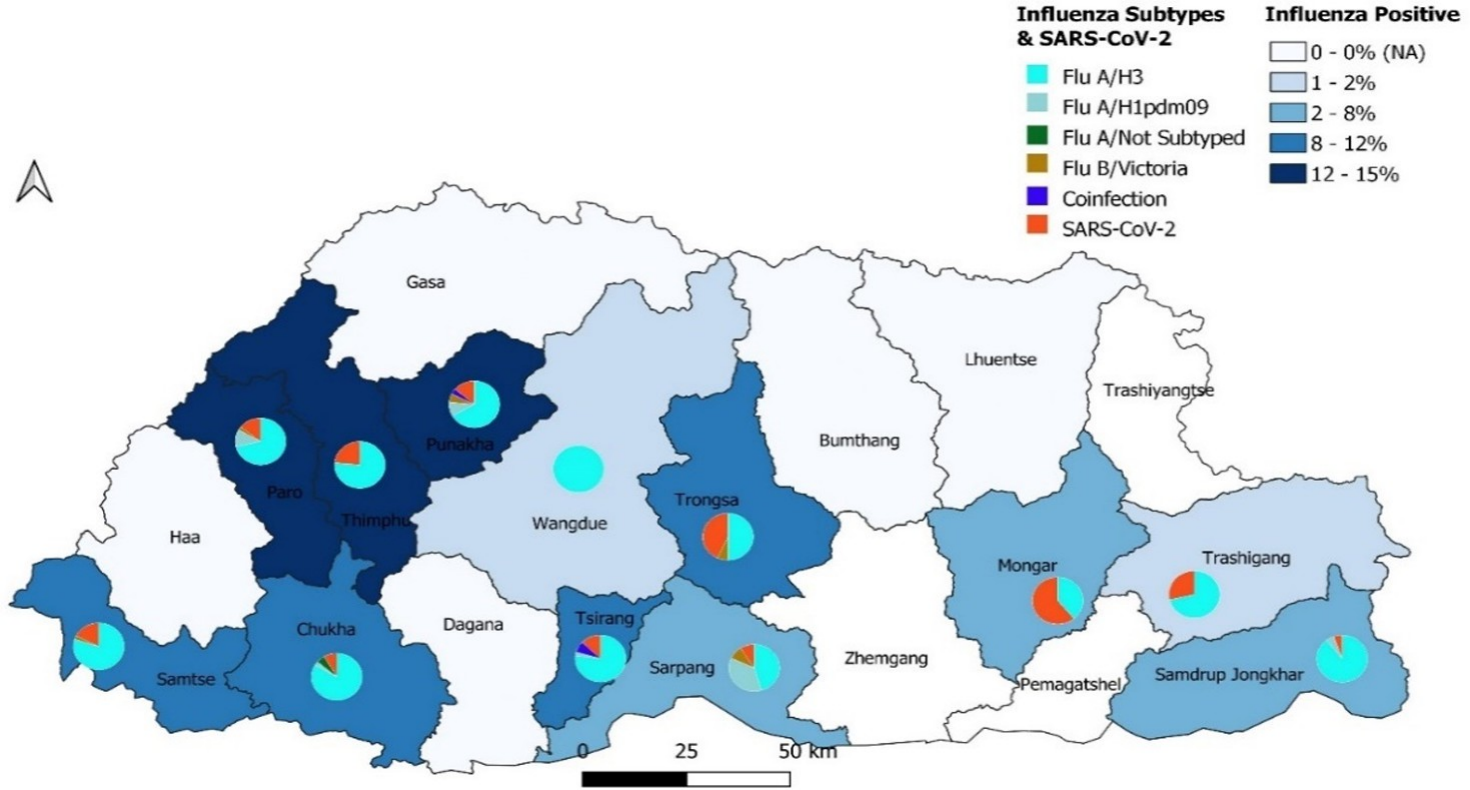
Distribution of Influenza subtypes and SARS-CoV-2 by districts.

The weekly average Flu positivity rate was 11.3% (0–64.5), and the positivity rate increased during epi weeks 41–48 (Oct–Dec 2022). The influenza positivity rate was higher in Paro, Thimphu, and Punakha districts (12–15%) compared to other sentinel hospitals, though it was not statistically significant (Fig 2).

The median age for flu among ILI cases was 12 years (IQR: 5–28) and among SARI was 6.2 (IQR: 2.5–15) years respectively. The most affected age group for flu among ILI was 15–29 years (29.4%) followed by 30–64 years (23.5%). Among the ILI cases Males (53.0%) were affected slightly more than females (47.0%) for flu, though statistically not significant (p-value: 0.51) (Table 3). Clinically the most common presenting symptoms for flu was cough (96.7%), fever (77.8%), and sore throat (64.8%) and the least was diarrhoea (3.3%) (Table 3).

**Table 3:** Patient demographic and clinical characteristics of influenza and SARS-CoV-2 positive ILI and SARI cases in Bhutan 2022.

	ILI					SARI			
Variables	All ILI n	Influenza n(%)	SARS-CoV-2 n(%)	Coinfection n(%)	P value	All SARI n	Influenza n(%)	SARS-CoV-2 n(%)	P value
Total	1373	270 (19.7)	65 (4.7)	5 (0.4)		473	10 (2.1)	9 (1.9)	
Median age (IQR), years	13 (6–29)	12 (5–28)	12 (5–26)	14 (8–54.5)		2.6 (1–29)	6.2 (2.5–15)	4 (1–63)	
Age group									
0–1 years	133	11 (8.3)	4 (3.0)	0	0.001	199	2 (1.0)	3 (1.5)	0.008
2–4 years	147	12 (8.2)	3 (2.0)	0		87	1 (1.1)	2 (2.3)	
5–14 years	438	77 (17.6)	8 (1.8)	3 (0.7)		45	5 (11.1)	0	
15–29 years	330	97 (29.4)	21 (6.4)	0		24	1 (4.2)	0	
30–64 years	293	69 (23.5)	24 (8.2)	2 (0.7)		50	0	2 (4.0)	
>65 years	32	4 (12.5)	5 (15.6)	0		68	1 (1.5)	2 (2.9)	
Sex									
Female	666	127 (47.0)	30 (46.2)	1 (20.0)	0.5	214	6 (60.0)	3 (33.3)	0.5
Male	707	143 (53.0)	35 (53.8)	4 (80.0)		259	4 (40.0)	6 (66.7)	
Clinical symptoms									
Fever	1125 (81.9)	210 (77.8)	53 (81.5)	3 (60.0)	0.12	425 (89.8)	9 (90.0)	7 (77.8)	0.4
Cough	1309 (95.3)	261 (96.7)	62 (95.4)	5 (100)	0.65	452 (95.6)	10 (100)	9 (100)	0.63
Sore throat	889 (64.8)	198 (73.3)	51 (78.5)	3 (60.0)	0.001	311 (65.8)	7 (70.0)	4 (44.4)	0.34
Breathing problem	192 (13.9)	55 (20.4)	9 (13.8)	1 (20.0)	0.008	271 (57.3)	5 (50.0)	5 (55.6)	0.88
Headache	719 (52.5)	169 (62.6)	30 (46.2)	3 (60.0)	0.002	110 (23.3)	1 (10.0)	9 (100)	0.145
Nausea/vomiting	98 (7.1)	18 (6.7)	1 (1.5)	0	0.27	51 (10.8)	1 (10.0)	0	0.57
Loss of Smell/taste	238 (17.3)	79 (29.3)	11 (16.9)	1 (20.0)	0.001	39 (8.3)	1 (10.0)	0	0.65
Chills	259 (18.9)	44 (16.3)	11 (16.9)	1 (20.0)	0.63	82 (17.3)	1 (10.0)	1 (11.1)	0.72
Muscle pain	370 (26.9)	125 (46.3)	16 (24.6)	3 (60.0)	0.001	45 (9.5)	3 (30.0)	0	0.05
Diarrhoea	60 (4.4)	9 (3.3)	1 (1.5)	0	0.43	38 (8.0)	2 (20.0)	0	0.25
Abdominal pain	114 (8.3)	24 (8.9)	5 (7.7)	0	0.83	32 (6.8)	1 (10.0)	0	0.66
Comorbidities									
Diabetes	1 (0.07)	0	0	0	0.95	10 (2.11)	0	0	0.12

### Viral genome characterization

NGS experiments were conducted on a total of 68 specimens from 2022, including 6 Flu A(H1N1)pdm09), 59 Flu A(H3N2), and 3 Flu B.

### Flu A(H1N1)pdm09 HA sequences analysis

**Fig 3:**
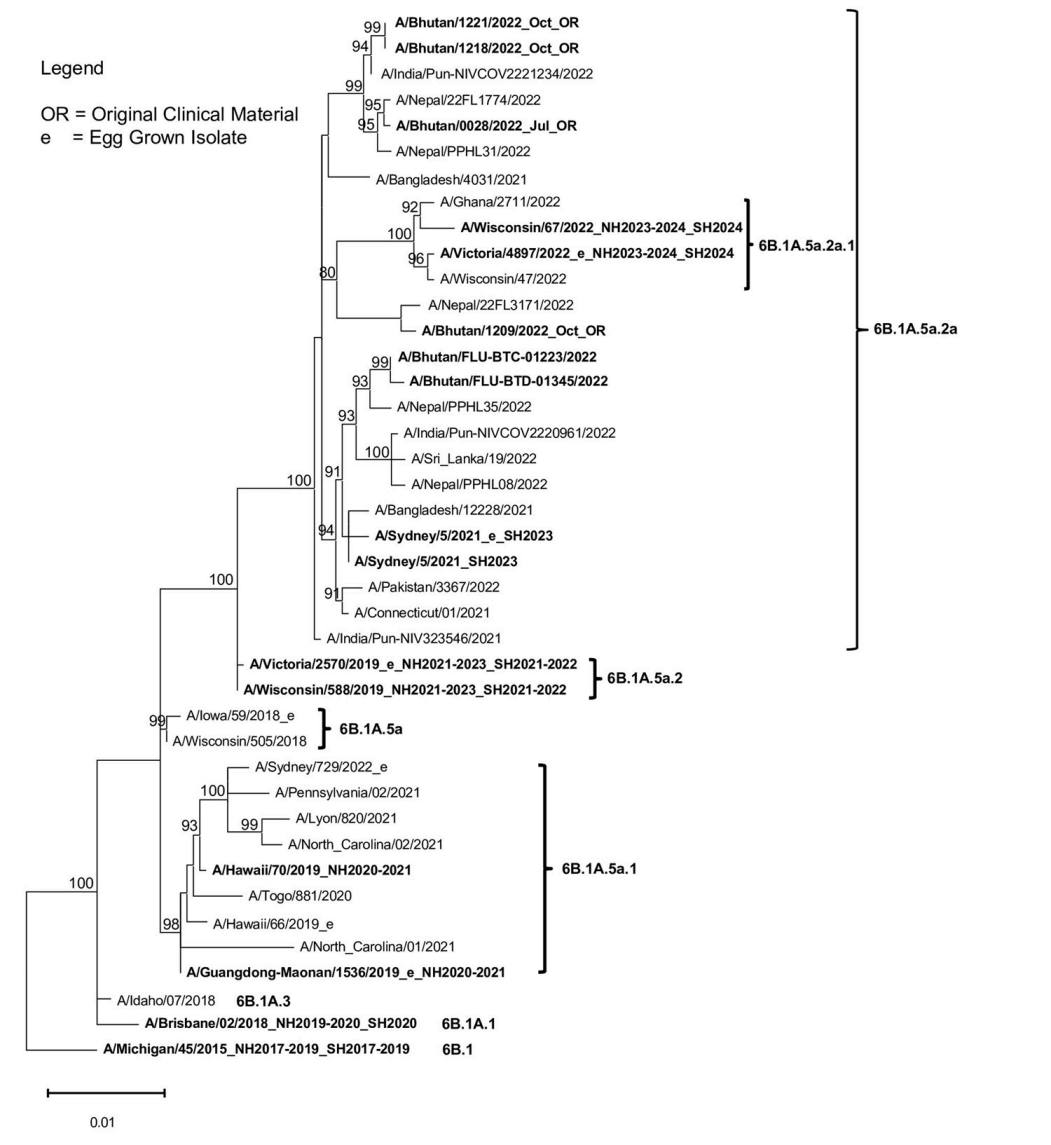
Maximum-likelihood tree of 41 Flu A(H1N1) pdm09 HA gene sequences (1,701 bases) comprised 6 sequences from viruses detected in Bhutan in 2022, alongside 35 sequences from the GISAID database including 10 vaccine strains and 14 representative sub-clades belonging to HA genetic clade 6B.1A.

The phylogenetic tree of Flu A(H1N1)pdm09 HA sequences revealed that all 6 A(H1N1)pdm09 viruses collected from July to November 2022 in Bhutan (6/17, 35%) belonged to clade 6B.1A.5a.2a. Notably, this subclade differed from the WHO-recommended vaccine strains A/Wisconsin/588/2019 and A/Victoria/2570/2019 used for the 2021 SH, 2021–2022 NH, 2022 SH, and 2022–2023 NH flu seasons which were classified under 6B.1A.5a.2 subclade. All Flu A (H1N1)pdm09 viruses from Bhutan in 2022 were however found to be clustered with A/Sydney/5/2021, which is one of the strains included in the composition of the Flu vaccine for the 2023 SH flu season (Fig 3).

When comparing the HA protein of the 2022 Bhutan Flu A (H1N1)pdm09 viruses with the A/Wisconsin/588/2019 vaccine strain (NH 2021–2023 and SH 2021–2022), 12 amino acid substitutions were identified in the HA1 subunit. These substitutions affected dominant epitopes: A141T in epitope A, I185V, A186T and Q189E in epitope B, K308R in epitope C, D94N, T216A, and E224A in epitope D, and A48P, K54Q, S83P, and R259K in epitope E (S3 File). Their impact on average VE was calculated using the *P*_epitope_ model, resulting in an efficacy estimate of 85.14% (E = 0.45 of 53%, *P*_epitope_ = 0) (Table 4).

**Table 4:** Vaccine effectiveness (VE) and substitutions in dominant epitopes of seasonal influenza virus circulating in Bhutan, 2022 compared to 2022 vaccine strains.

Subtype	Vaccine Strain	No. Strains	Dominant epitope	No. of substitution	*P*epitope	Efficacy	E (%)	E (100%)
Flu A(H1N1)pdm09 (n = 6)	A/Wisconsin/588/2019	0	NO	0	0.000	0.530	53.00	100.00
		2	A	1	0.042	0.480	48.04	90.64
		6	B	2	0.091	0.422	42.18	79.59
		1	B	3	0.136	0.368	36.77	69.38
		6	C	1	0.030	0.494	49.39	93.20
		4	D	1	0.021	0.505	50.52	95.32
		2	D	3	0.063	0.456	45.56	85.97
		5	E	3	0.088	0.425	42.50	80.19
		1	E	2	0.059	0.460	46.00	86.79
				Average *P*epitope	0.066	0.4512	45.12	85.14
Flu A(H3N2) (n = 59)	A/Darwin/9/2021	0	NO	0	0.00	0.47	47.00	100.00
		36	A	1	0.053	0.340	34.00	72.34
		1	A	2	0.105	0.210	21.00	44.68
		59	B	2	0.095	0.235	23.48	49.95
		54	C	1	0.037	0.379	37.85	80.54
		5	C	2	0.074	0.287	28.70	61.07
		40	D	1	0.024	0.410	40.98	87.18
		19	D	2	0.049	0.350	34.95	74.36
		37	E	1	0.045	0.358	35.77	76.11
				Average *P*epitope	0.060	0.321	32.09	68.28
Flu B (Vitoria, n = 3)	B/Washington/02/2019	0	NO	0	0.000	0.682	68.24	100.00
		3	A	2	0.091	0.604	60.39	88.49
		3	B	4	0.167	0.538	53.84	69.82
		3	D	1	0.025	0.661	66.08	67.61
				Average *P*epitope	0.094	0.601	60.10	88.07

### Flu A(H3N2) HA sequences analysis

**Fig 4:**
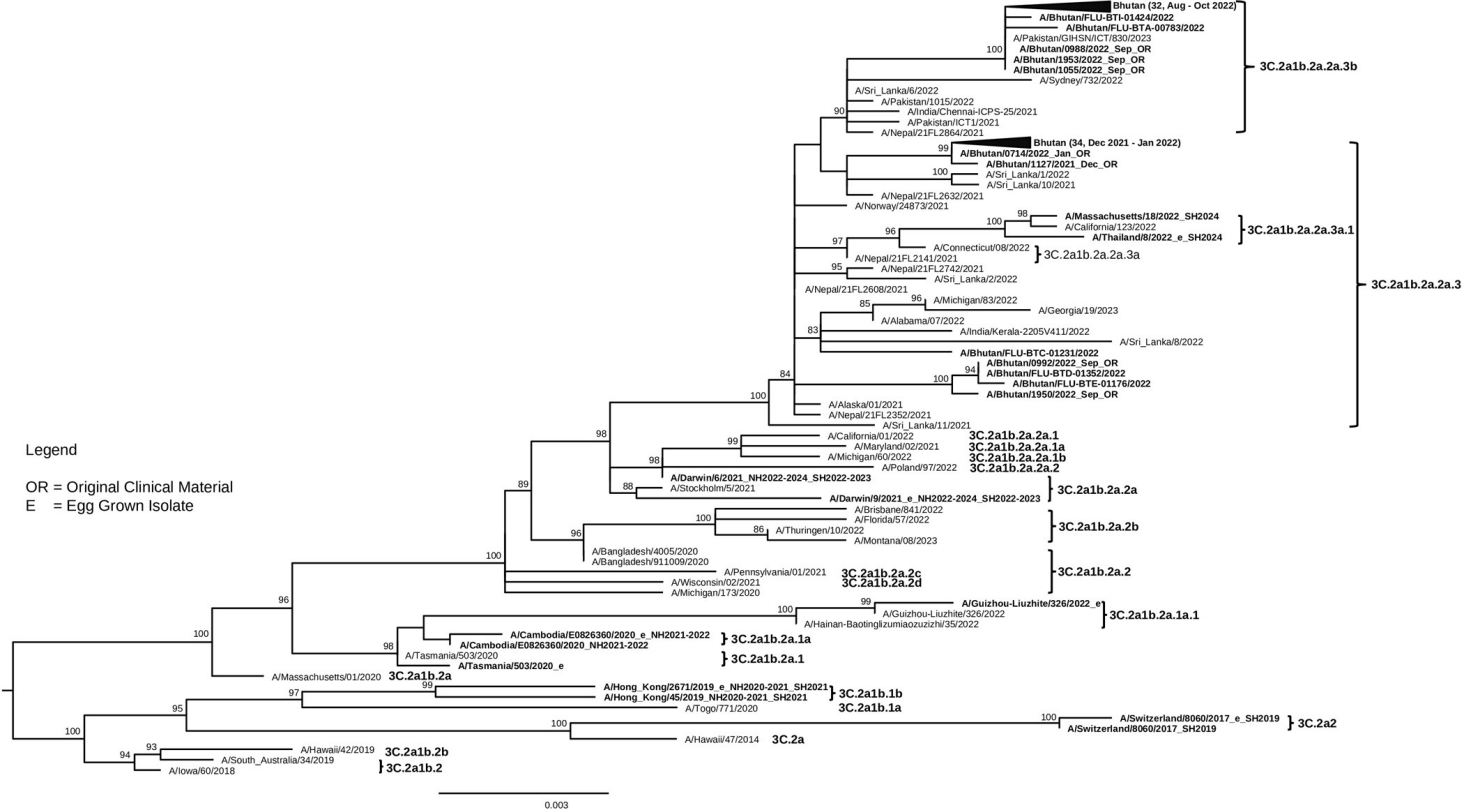
Maximum-likelihood tree of 138 Flu A(H3N2) HA gene sequences (1,701 bases) comprised 78 sequences from viruses detected in Bhutan in 2021 (19 sequences) and 2022 (59), alongside with 60 sequences from the GISAID database including 10 vaccine strains and 33 representative sub-clades belonging to HA genetic clade 3C.2A.

The phylogenetic tree of Flu A(H3N2) HA sequences revealed that all 59 A(H3N2) viruses from Bhutan in 2022 (59/252, 23%) were in the same clade 3C.2a1b.2a but fell into two different subclades: subclade 2a.3, which accounted for the majority (39/59, 66%) and included viruses collected from January to December 2022, and subclade 2a.3b, which constituted the remaining (22/59, 37%) and included viruses collected from August to December 2022. These identified subclades were different from the vaccine stains including A/Cambodia/e0826360/2020 (subclade 3C.2a1b.2a.1a) used for the 2021–2022 NH flu season, and A/Darwin/6/2021 (subclade 3C.2a1b.2a.2a) and A/Darwin/9/2021 (subclade 3C.2a1b.2a.2a) used for the 2022 SH, 2022–2023 NH, 2023 SH, and 2023–2024 NH flu season (Fig 4).

When comparing the HA protein of the 2022 Bhutan Flu A (H3N2) viruses with the A/Darwin/9/2021 vaccine strain (NH 2022–2024 and SH 2022–2023), 16 amino acid substitutions were identified in the HA1 subunit. Among these 12 substitutions affected dominant epitopes: S124I and I140M in epitope A, N186D and I192F in epitope B, I48T, D53N/S and V309I in epitope C, N96S, P103Q, I214T and S219Y in epitope D, and E83K in epitope E (S3 File). Their impact on average VE was calculated using the *P*_epitope_ model, resulting in an efficacy estimate of 68.28% (E = 0.32 of 47%, *P*_epitope_ = 0) (Table 4). Additionally, other substitution in HA1 subunit include N6D, T30I, G78D and G225D. Five substitutions were found in the HA2 subunit: N49S, S113A, I149M, V176I and G181E.

### Flu B HA sequences analysis

**Fig 5:**
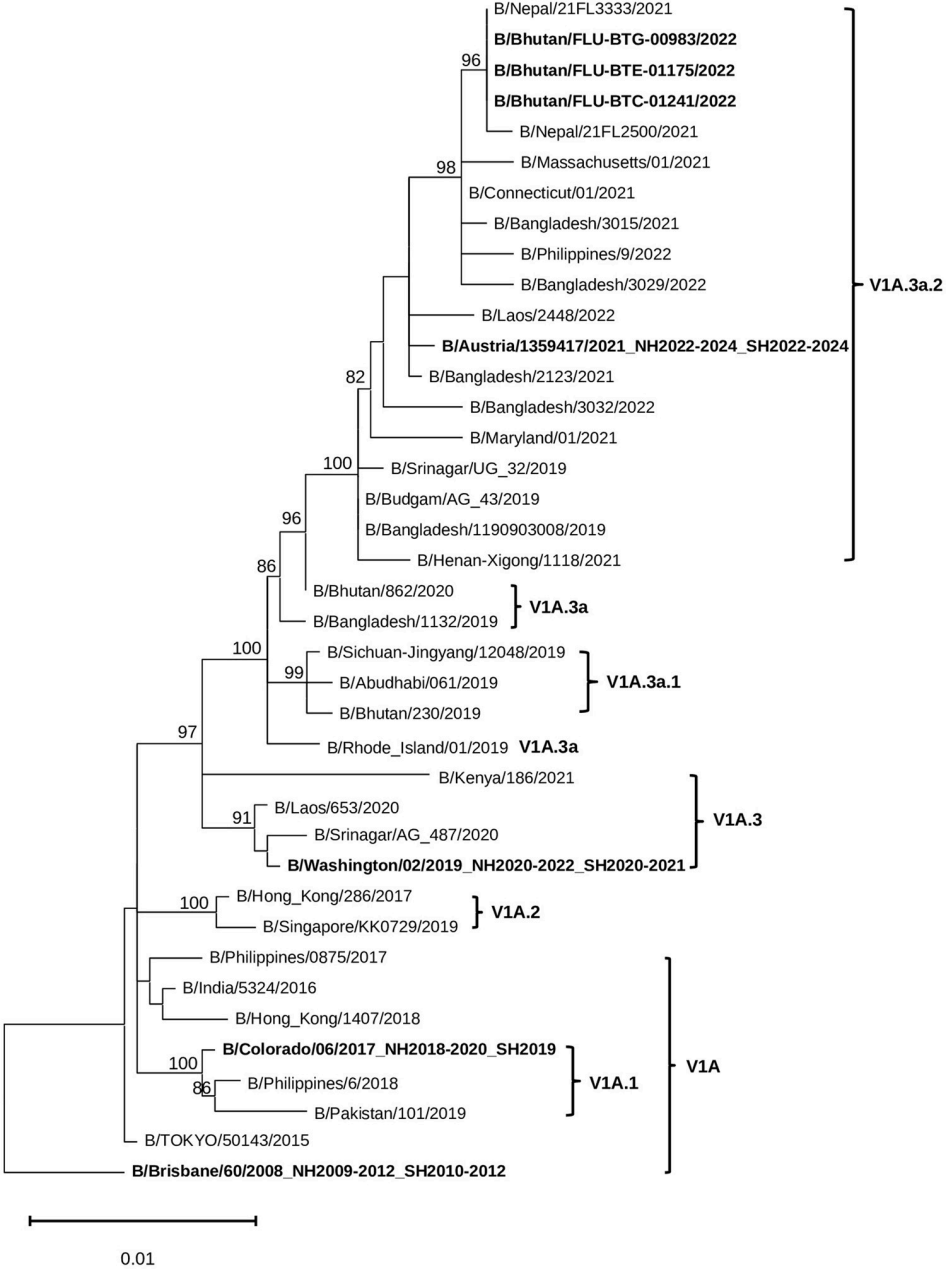
Maximum-likelihood tree of 39 Flu B HA gene sequences (1,758 bases) comprised 3 sequences from viruses detected in Bhutan in 2022, alongside 36 sequences from the GISAID database including 4 vaccine strains and 32 representative sub-clades belonging to HA genetic clade V1A.

The phylogenetic tree of Flu B HA sequences revealed that all 3 Flu B/Victoria viruses from Bhutan in the year 2022 (3/9, 33%), collected from November to December belonged to subclade V1A.3a.2 and clustered together with the vaccine strain used in Bhutan, Austria/1359417/2021 (2022–2023 NH, 2022 SH, 2023 SH and 2024 SH flu season) (Fig 5).

When comparing the HA protein of the 2022 Bhutan Flu B viruses with the B/Washigton/02/2019 vaccine strain (NH 2020–2022 and SH 2020–2021), 8 amino acid substitutions were identified in the HA1 domain. Among these 7 substitutions affected dominant epitopes: P144L and N150K in epitope A, A127T, R133G, S196E and K202R in epitope B, and G183E in epitope D (S3 File). Their impact on average VE was calculated using the *P*_epitope_ model, resulting in an efficacy estimate of 88.07% (E = 0.60 of 68.24%, *P*_epitope_ = 0) (Table 4). Additionally, 1 amino acid substitutions, R278K, was found in the HA1 domain.

### Antiviral drug resistance

Results from phenotypic assay showed that 11 isolates of Flu A(H3N2) were susceptible to oseltamivir (Ose), zanamivir (Zan), peramivir (Per), and laninamivir (Lan) by antiviral assay. For investigating mutations in NA gene sequences associated with Ose resistance, we found that none of the Flu virus sequences in this study exhibited markers of resistance to NA inhibitors in NA.

### Antigenic characterization

Of the three Flu A(H3N2) viruses antigenically characterized by HINT, one subclade of 3C.2a1b.2a.2a.3b was well-recognized to A/DARWIN/6/2021-LIKE (H3N2) by HINT when ferret antisera was raised against cell-propagated reference strain, and two subclades of 3C.2a1b.2a.2a.3 were recognized to A/CAMBODIA/E0826360/2020-LIKE (H3N2) low by HINT when ferret antisera was raised against cell-propagated reference strain.

## Discussion

During the 2021–2022 NH, and SH Flu seasons, Flu A(H1N1)pdm09, A(H3N2), and B/Victoria viruses co-circulated, although the proportions of the viruses circulating and the timing of activity varied among countries and regions [31–33].

Based on weekly monitoring of Flu positivity rates, Bhutan experienced significant Flu activity in 2022 compared to 2020 and 2021, especially from October to December, despite the COVID-19 pandemic considerably disrupting surveillance for the last two years [34]. Flu positivity was substantially increased (15.2%) with Flu A(H3N2) (70.2%) being the most predominant strain during the year. Globally, Flu activity remained elevated due to activity in the NH. In cases where subtyping was performed, Flu A viruses were the predominant strain, with a slightly higher proportion of A(H3N2) viruses identified among the subtyped Flu A viruses [35]. India, a neighbouring country sharing border with Bhutan however reported Flu A/H1N1pdm09 as the predominant circulating strain [36].

In the Northern Hemisphere (NH), the flu activity typically peaks during the winter months, which span from December to end of March. Conversely, the Southern Hemisphere (SH) experiences its Flu season between July and August [37]. Bhutan however has two distinct Flu seasons; one during winter and another during the monsoon season [7]. This dual pattern can be attributed to the differential impact of absolute humidity and temperature on the virus: low humidity enhances viral stability in colder conditions, while high humidity aids in viral survival in warmer conditions [38, 39]. Another reason could be the reopening of schools in February, which is a significant factor in the increase of Flu cases due to the dense gatherings of students and staff in educational settings.

Similar to previous studies, we found the median age of Flu among ILI was higher than SARI. The median age for Flu among ILI was 12 years (5–28 years), while the median age for Flu among SARI was 6.2 years (2.5–15 years). In addition, the most affected age group by Flu was 15–29 years (35.9%) followed by 5–14 years (28.5%) which is also consistent with the higher Flu morbidity rates in younger ages discussed in prior studies [40].

The viral genomic data from this study provide valuable insights into the dynamics of Flu viruses and their implications for vaccine efficacy and viral circulation in Bhutan. HA sequences of Flu A(H1N1)pdm09 analyzed in this study were obtained from viruses that represented 35% of all detected Flu A(H1N1)pdm09 viruses, belonging to subclade 6B.1A.5a.2a. It was previously reported that this subclade was predominating in Asia and some countries in Europe and Africa during September 2022 to February 2023 [41]. This subclade differed from the WHO-recommended vaccine strains for the 2021–2022 NH Flu season and 2022 SH Flu season. This discrepancy highlights potential concerns regarding the effectiveness of the 2021–2022 vaccine against the circulating viruses. In addition, regarding antigenic characterization of Flu A(H1N1)pdm09, it was previously reported that Ferret antisera did not detect large antigenic differences between the viruses with 5a.2 and 5a.2a HA genes [41].

Similarly, we observed that HA sequences from Flu B viruses (collected from November to December) accounted for 33% of all detected Flu B viruses in this study. These sequences belonged to subclade V1A.3a.2, which was distributed globally from September 2022 to January 2023 [40]. This subclade matched with B/Austria/1359417/2021 (2022–2023 NH Flu vaccine strain), one of the strains included in Flu vaccine available for 2022 in Bhutan. Post-infection ferret antisera raised against B/Austria/1359417/2021-like viruses (2022–2024 NH and SH vaccine stain) were previously reported to effectively inhibit HA clade 1A.3a.2 viruses [41]. This finding suggests that there is good efficacy of the Flu vaccine used during Bhutan’s 2022–2023 Flu season against the circulating Flu B strains.

For Flu A(H3N2) viruses, the major subtype that circulated in 2022, HA sequences obtained from viruses representing 23% of all detected Flu A(H3N2) viruses in this study were divided into two subclades: 3C.2a1b.2a.2a.3 (which circulated from January to December) and 3C.2a1b.2a.2a.3b (which circulated from August to December). Surprisingly, subclades 2a.1b, 2a.3a.1, and 2b were circulating in many countries from September 2022 to February 2023, especially subclade 2b, which predominated in this period [41], but they were not detected in Bhutan. This points to significant geographic variation in viral distribution, raising questions about factors influencing these patterns, such as human movement, local immunity levels, and environmental conditions. The phylogenetic tree also revealed that subclade 3C.2a1b.2a.2a.3 has been circulating in Bhutan since 2021 and is closely related to two reference viruses from 2021: A/Alaska/01/2021 and A/Norway/24873/2021. As for subclade 3C.2a1b.2a.2a.3b, it was first detected in Bhutan in 2022 and is closely related to the reference virus A/Sydney/732/2022. Notably, these subclades differed from the vaccine strains used in the NH 2021–2024 and SH 2022–2023 seasons. This discrepancy raises concerns about the effectiveness of vaccination in protecting against the outbreak of Flu A(H3N2) in Bhutan (VE = 68.28%). While the previous report indicated that multiple serum panels from subjects vaccinated with A/Darwin/6/2021-like viruses exhibited good neutralization against viruses expressing various emerging 2a subclade HA genes, including 2a.3 subclade HA gene, the results for neutralization against viruses expressing 2a.3b were not available [41].

In addition, the presence of several amino acid substitutions in the HA protein of the circulating viruses investigated in this study compared to the vaccine strains underscores the ongoing evolution of the virus, which could potentially impact vaccine efficacy. Fortunately, the 11 Flu A(H3N2) virus isolates in our study yielded negative NAI results. Additionally, no mutations associated with oseltamivir resistance were found in the viruses isolated from this study, despite testing a small number of samples, which included 11 Flu A(H3N2) viruses tested by NAI and 68 Flu NA sequences (comprising 35% of Flu A(H1N1)pdm09, 23% of Flu A(H3N2), and 33% of Flu B). These findings suggest the potential effectiveness of using oseltamivir during the outbreak.

In Bhutan seasonal Flu vaccine was initiated in 2019 by the Expanded Immunization program (now the Vaccine Preventable Division program) under the Ministry of Health, and rolled out to 5 high-risk groups of the population. The vaccine coverage in 2022 for these high-risk groups was 87.2%, 70.8%, 60.8%, 87.9%, and 93.3% for children 6 to less than 24 months, elderly >65 years and above, pregnant women, health workers, and population with existing chronic medical conditions, respectively [6]. Although the vaccination program reflected successful efforts in protecting vulnerable populations in this country, findings from this study demonstrate the dynamic nature of Flu viruses and the necessity of vigilant surveillance and vaccine strain selection to ensure optimal protection against the ever-evolving Flu strains.

### Limitation

First, surveillance was disrupted by the COVID-19 pandemic due to health workers’ involvement in the pandemic response, and therefore, consistent data reporting was not available from all sites. Second, training on ILI and SARI guideline couldn’t be conducted during the COVID-19 pandemic which has affected the health workers’ participation and case enrolment from sentinel sites. Third, Flu positive specimens shipment from the sentinel sites to RCDC and then to the WHO Collaborating Centre (WHO-CC) was challenging due to cold-chain logistic issues.

## Conclusion

Flu positivity rates were significantly elevated in Bhutan during the Flu season in 2022. Flu A(H3N2) subtype was the predominant circulating strain in the country and globally. Genetic characterization of the Flu viruses in Bhutan showed close relatedness of high vaccine efficacy with the vaccine strain that WHO recommended for the 2022–23 season. Sustaining robust Flu surveillance is key to contributing significantly towards the influenza vaccine recommendations made each year by WHO.

## Supporting information

S1 File Gene accession number. (XLSX)

S2 File Acknowledgment list and reference gene accession number. (XLSX)

S3 File The alignment of HA protein sequences of seasonal influenza viruses in Bhutan in 2022. (DOCX)

S4 File Datasets. (XLSX)
