# Supplementary material for: Epidemiology and genetic characterization of influenza viruses circulating in Bhutan in 2022
Source: PLoS One. 2024 Sep 17;19(9):e0304849. doi: 10.1371/journal.pone.0304849 (PMC11407632; doi:10.1371/journal.pone.0304849)
Supplement: S3 File — (DOCX) [file pone.0304849.s003.docx]

**Supplementary (S3). The alignment of HA protein sequences of seasonal influenza viruses in Bhutan in 2022. Amino acid substitutions between the vaccine strain (show in bold) and the circulating strains from Bhutan in 2022 were observed in:**

A) Flu A (H1N1)pdm09 compared to A/Wisconsin/588/2019 (accession no. EPI1661231)

B) Flu A (H3N2) compared to A/Darwin/09/2021 (accession no. EPI1859996)

C) Flu B (Victoria-like lineage) compared to B/Washington/02/2019 (accession no. MN155753.1)

**A)**

| **Flu A (H1N1)pdm 09** | | **HA1** | | | | | | | | | | | |
| --- | --- | --- | --- | --- | --- | --- | --- | --- | --- | --- | --- | --- | --- |
| Clade | **Epitope** | **E** | **E** | **E** | **D** | **A** | **B** | **B** | **B** | **D** | **D** | **E** | **C** |
|  | **Amino acid position** | **48** | **54** | **83** | **94** | **141** | **185** | **186** | **189** | **216** | **224** | **259** | **308** |
| **6B.1A.5a.2** | **A/Wisconsin/588/2019** | **A** | **K** | **S** | **D** | **A** | **I** | **A** | **Q** | **T** | **E** | **R** | **K** |
| 6B.1A.5a.2a | A/Bhutan/0028/2022 | P | Q | - | - | - | - | T | E | - | A | K | R |
|  | A/Bhutan/1209/2022 | - | Q | - | - | - | V | T | E | - | A | K | R |
|  | A/Bhutan/1218/2022 | P | Q | - | - | - | - | T | E | - | A | K | R |
|  | A/Bhutan/1221/2022 | P | Q | - | - | - | - | T | E | - | A | K | R |
|  | A/Bhutan/FLU-BTC-01223/2022 | - | Q | P | N | T | - | T | E | A | A | K | R |
|  | A/Bhutan/FLU-BTD-01345/2022 | - | Q | P | N | T | - | T | E | A | A | K | R |

**B)**

| **Flu A (H3N2)** | | **HA1** | | | | | | | | | | | | | | | | **HA2** | | | | |
| --- | --- | --- | --- | --- | --- | --- | --- | --- | --- | --- | --- | --- | --- | --- | --- | --- | --- | --- | --- | --- | --- | --- |
| Clade | **Epitope** |  |  | **C** | **C** |  | **E** | **D** | **D** | **A** | **A** | **B** | **B** | **D** | **D** |  | **C** |  |  |  |  |  |
|  | **Amino acid position** | **6** | **30** | **48** | **53** | **78** | **83** | **96** | **103** | **124** | **140** | **186** | **192** | **214** | **219** | **225** | **309** | **49** | **113** | **149** | **176** | **181** |
| **3C.2a1b.2a.2a** | **A/Darwin/09/2021** | **N** | **T** | **I** | **D** | **G** | **E** | **N** | **P** | **S** | **I** | **N** | **I** | **I** | **S** | **G** | **V** | **N** | **S** | **I** | **V** | **G** |
| 3C.2a1b.2a.2a.3 | A/Bhutan/0437/2022 | - | - | - | S | - | - | S | - | - | - | D | F | T | - | D | - | S | - | - | - | - |
| 3C.2a1b.2a.2a.3 | A/Bhutan/FLU-BTE-00864/2022 | - | - | - | N | - | - | S | - | - | - | D | F | T | - | D | - | S | - | - | - | - |
| 3C.2a1b.2a.2a.3 | A/Bhutan/1253/2022 | - | - | - | N | - | - | S | - | - | - | D | F | T | - | D | - | S | - | - | - | - |
| 3C.2a1b.2a.2a.3 | A/Bhutan/0442/2022 | - | - | - | N | - | - | S | - | - | - | D | F | T | - | D | - | S | - | - | - | - |
| 3C.2a1b.2a.2a.3 | A/Bhutan/1254/2022 | - | - | - | N | - | - | S | - | - | - | D | F | T | - | D | - | S | - | - | - | - |
| 3C.2a1b.2a.2a.3 | A/Bhutan/1255/2022 | - | - | - | N | - | - | S | - | - | - | D | F | T | - | D | - | S | - | - | I | - |
| 3C.2a1b.2a.2a.3 | A/Bhutan/1256/2022 | - | - | - | N | - | - | S | - | - | - | D | F | T | - | D | - | S | - | - | - | - |
| 3C.2a1b.2a.2a.3 | A/Bhutan/1149/2022 | - | - | - | N | - | - | S | - | - | - | D | F | T | - | D | - | S | - | - | - | - |
| 3C.2a1b.2a.2a.3 | A/Bhutan/FLU-BTC-01151/2022 | - | - | - | N | - | - | S | - | - | - | D | F | T | - | D | - | S | - | - | - | - |
| 3C.2a1b.2a.2a.3 | A/Bhutan/1318/2022 | - | - | - | N | - | - | S | - | - | - | D | F | T | - | D | - | S | - | - | - | - |
| 3C.2a1b.2a.2a.3 | A/Bhutan/1257/2022 | - | - | - | N | - | - | S | - | - | - | D | F | T | - | D | - | S | - | - | - | - |
| 3C.2a1b.2a.2a.3 | A/Bhutan/1258/2022 | - | - | - | N | - | - | S | - | - | - | D | F | T | - | D | - | S | - | - | - | - |
| 3C.2a1b.2a.2a.3 | A/Bhutan/0452/2022 | - | - | - | N | - | - | S | - | - | - | D | F | T | - | D | - | S | - | - | - | - |
| 3C.2a1b.2a.2a.3 | A/Bhutan/FLU-BTE-00878/2022 | - | - | - | N | D | - | S | - | - | - | D | F | T | - | D | - | S | - | - | - | - |
| 3C.2a1b.2a.2a.3 | A/Bhutan/0456/2022 | - | I | - | N | - | - | S | - | - | - | D | F | T | - | D | - | S | - | - | - | - |
| 3C.2a1b.2a.2a.3 | A/Bhutan/FLU-BTD-01270/2022 | - | - | - | N | - | - | S | - | - | - | D | F | T | - | D | - | S | - | - | - | - |
| 3C.2a1b.2a.2a.3 | A/Bhutan/0714/2022 | - | - | - | N | - | - | S | - | - | - | D | F | T | - | D | - | S | - | - | - | - |
| 3C.2a1b.2a.2a.3b | A/Bhutan/1047/2022 | - | - | - | N | - | K | S | - | - | M | D | F | - | - | D | - | S | A | M |  |  |
| 3C.2a1b.2a.2a.3b | A/Bhutan/1051/2022 | - | - | - | N | - | K | S | - | - | M | D | F | - | - | D | - | S | A | M | - | - |
| 3C.2a1b.2a.2a.3b | A/Bhutan/1052/2022 | - | - | - | N | - | K | S | - | - | M | D | F | - | - | D | - | S | A | M | - | - |
| 3C.2a1b.2a.2a.3b | A/Bhutan/1055/2022 | - | - | - | N | - | K | S | - | - | M | D | F | - | - | D | - | S | A | M | - | - |
| 3C.2a1b.2a.2a.3b | A/Bhutan/1057/2022 | - | - | - | N | - | K | S | - | - | M | D | F | - | - | D | - | S | A | M | - | - |
| 3C.2a1b.2a.2a.3b | A/Bhutan/0980/2022 | - | - | - | N | - | K | S | - | - | M | D | F | - | - | D | - | S | A | M | - | - |
| 3C.2a1b.2a.2a.3b | A/Bhutan/0988/2022 | - | - | - | N | - | K | S | - | - | M | D | F | - | - | D | - | S | A | M | - | - |
| 3C.2a1b.2a.2a.3b | A/Bhutan/0991/2022 | - | - | - | N | - | K | S | - | - | M | D | F | - | - | D | - | S | A | M | - | - |
| 3C.2a1b.2a.2a.3 | A/Bhutan/0992/2022 | D | - | T | N | - | - | S | - | - | - | D | F | - | - | D | - | S | - | - | - | - |
| 3C.2a1b.2a.2a.3b | A/Bhutan/0994/2022 | - | - | - | N | - | K | S | - | - | M | D | F | - | - | D | - | S | A | M | - | - |
| 3C.2a1b.2a.2a.3b | A/Bhutan/0996/2022 | - | - | - | N | - | K | S | - | - | M | D | F | - | - | D | - | S | A | M | - | - |
| 3C.2a1b.2a.2a.3b | A/Bhutan/1001/2022 | - | - | - | N | - | K | S | - | - | M | D | F | - | - | D | - | S | A | M | - | - |
| 3C.2a1b.2a.2a.3b | A/Bhutan/1002/2022 | - | - | - | N | - | K | S | - | - | M | D | F | - | - | D | - | S | A | M | - | - |
| 3C.2a1b.2a.2a.3b | A/Bhutan/FLU-BTI-01352/2022 | - | - | - | N | - | K | S | - | - | M | D | F | - | - | D | - | S | A | M | - | - |
| 3C.2a1b.2a.2a.3b | A/Bhutan/1287/2022 | - | - | - | N | - | K | S | - | - | M | D | F | - | - | D | - | S | A | M | - | - |
| 3C.2a1b.2a.2a.3 | A/Bhutan/1950/2022 | D |  | T | N | - | - | S | Q | - | - | D | F | - | - | D | - | S | - | - | - | - |
| 3C.2a1b.2a.2a.3b | A/Bhutan/1953/2022 | - | - | - | N | - | K | S | - | - | M | D | F | - | - | D | - | S | A | M | - | - |
| 3C.2a1b.2a.2a.3b | A/Bhutan/1955/2022 | - | - | - | N | - | K | S | - | - | M | D | F | - | - | D | - | S | A | M | - | - |
| 3C.2a1b.2a.2a.3b | A/Bhutan/1956/2022 | - | - | - | N | - | K | S | - | - | M | D | F | - | - | D | - | S | A | M | - | - |
| 3C.2a1b.2a.2a.3b | A/Bhutan/1360/2022 | - | - | - | N | - | K | S | - | - | M | D | F | - | - | D | - | S | A | M | - | - |
| 3C.2a1b.2a.2a.3b | A/Bhutan/1366/2022 | - | - | - | N | - | K | S | - | - | M | D | F | - | - | D | - | S | A | M | - | - |
| 3C.2a1b.2a.2a.3b | A/Bhutan/1962/2022 | - | - | - | N | - | K | S | - | - | M | D | F | - | - | D | - | S | A | M | - | - |
| 3C.2a1b.2a.2a.3b | A/Bhutan/1372/2022 | - | - | - | N | - | K | S | - | - | M | D | F | - | - | D | - | S | A | M | - | - |
| 3C.2a1b.2a.2a.3b | A/Bhutan/1374/2022 | - | - | - | N | - | K | S | - | - | M | D | F | - | - | D | - | S | A | M | - | - |
| 3C.2a1b.2a.2a.3b | A/Bhutan/1966/2022 | - | - | - | N | - | K | S | - | - | M | D | F | - | - | D | - | S | A | M | - | - |
| 3C.2a1b.2a.2a.3b | A/Bhutan/1971/2022 | - | - | - | N | - | K | S | - | - | M | D | F | - | - | D | - | S | A | M | - | - |
| 3C.2a1b.2a.2a.3b | A/Bhutan/1973/2022 | - | - | - | N | - | K | S | - | - | M | D | F | - | - | D | - | S | A | M | - | - |
| 3C.2a1b.2a.2a.3b | A/Bhutan/1306/2022 | - | - | - | N | - | K | S | - | - | M | D | F | - | - | D | - | S | A | M | - | - |
| 3C.2a1b.2a.2a.3b | A/Bhutan/FLU-BTA-00748/2022 | - | - | - | N | - | K | S | - | - | M | D | F | - | - | D | I | S | A | M | - | E |
| 3C.2a1b.2a.2a.3b | A/Bhutan/FLU-BTC-01222/2022 | - | - | - | N | - | K | S | - | - | M | D | F | - | - | D | - | S | A | M | - | - |
| 3C.2a1b.2a.2a.3b | A/Bhutan/FLU-BTI-01411/2022 | - | - | - | N | - | K | S | - | - | M | D | F | - | - | D | - | S | A | M | - | - |
| 3C.2a1b.2a.2a.3b | A/Bhutan/FLU-BTD-01326/2022 | - | - | - | N | - | K | S | - | - | M | D | F | - | - | D | - | S | A | M | - | - |
| 3C.2a1b.2a.2a.3b | A/Bhutan/FLU-BTH-00738/2022 | - | - | - | N | - | K | S | - | - | M | D | F | - | - | D | - | S | A | M | - | - |
| 3C.2a1b.2a.2a.3b | A/Bhutan/FLU-BTD-01342/2022 | - | - | - | N | - | K | S | - | - | M | D | F | - | - | D | - | S | A | M | - | - |
| 3C.2a1b.2a.2a.3b | A/Bhutan/FLU-BTI-01424/2022 | - | - | - | N | - | K | S | - | - | M | D | F | - | - | D | - | S | A | M | - | - |
| 3C.2a1b.2a.2a.3 | A/Bhutan/FLU-BTC-01231/2022 | - | - | - | N | - | - | S | - | - | - | D | F | - | - | D | - | S | - | - | - | - |
| 3C.2a1b.2a.2a.3 | A/Bhutan/FLU-BTD-01352/2022 | D | - | T | N | - | - | S | - | - | - | D | F | - | - | D | - | S | - | - | - | - |
| 3C.2a1b.2a.2a.3b | A/Bhutan/FLU-BTG-00988/2022 | - | - | - | N | - | K | S | - | - | M | D | F | - | - | D | - | S | A | M | - | - |
| 3C.2a1b.2a.2a.3b | A/Bhutan/FLU-BTE-01158/2022 | - | - | - | N | - | K | S | - | - | M | D | F | - | - | D | - | S | A | M | - | - |
| 3C.2a1b.2a.2a.3b | A/Bhutan/FLU-BTA-00783/2022 | - | - | - | N | - | K | S | - | I | M | D | F | - | Y | D | - | S | A | M | - | - |
| 3C.2a1b.2a.2a.3 | A/Bhutan/FLU-BTE-01176/2022 | D | - | T | N | - | - | S | - | - | - | D | F | - | - | D | - | S | - | - | - | - |
| 3C.2a1b.2a.2a.3b | A/Bhutan/FLU-BTI-01436/2022 | - | - | - | N | - | K | S | - | - | M | D | F | - | - | D | - | S | A | M | - | - |

**C)**

| **Flu B (Victoria)** | | **HA1** | | | | | | | |
| --- | --- | --- | --- | --- | --- | --- | --- | --- | --- |
| Clade | **Epitope** | **B** | **B** | **A** | **A** | **D** | **B** | **B** |  |
|  | **Amino acid position** | **127** | **133** | **144** | **150** | **183** | **196** | **202** | **278** |
| **V1A.3** | **B/Washington/02/2019** | **A** | **R** | **P** | **N** | **G** | **S** | **K** | **R** |
| V1A.3a.2 | B/Bhutan/FLU-BTC-01241/2022 | T | G | L | K | E | E | R | K |
| V1A.3a.2 | B/Bhutan/FLU-BTE-01175/2022 | T | G | L | K | E | E | R | K |
| V1A.3a.2 | B/Bhutan/FLU-BTG-00983/2022 | T | G | L | K | E | E | R | K |
